# MicroRNAs in toxic acute kidney injury: Systematic scoping review of the current status

**DOI:** 10.1002/prp2.695

**Published:** 2021-02-18

**Authors:** Fathima Shihana, Melissa L. Barron, Fahim Mohamed, Devanshi Seth, Nicholas A. Buckley

**Affiliations:** ^1^ Clinical Pharmacology and Toxicology Research Group Discipline of Pharmacology Faculty of Medicine and Health The University of Sydney Sydney NSW Australia; ^2^ South Asian Clinical Toxicology of Research Collaboration Faculty of Medicine University of Peradeniya Peradeniya Sri Lanka; ^3^ Department of Pharmacy Faculty of Allied Health Sciences University of Peradeniya Peradeniya Sri Lanka; ^4^ Discipline of Clinical Medicine & Addiction Medicine Faculty of Medicine and Health The University of Sydney Sydney NSW Australia; ^5^ Drug Health Services Royal Prince Alfred Hospital Camperdown NSW Australia; ^6^ The Centenary Institute of Cancer Medicine & Cell Biology The University of Sydney Sydney NSW Australia

**Keywords:** acute kidney injury, biomarkers, circulating microRNA, nephrotoxicity, urinary microRNAs

## Abstract

Acute kidney injury induced by nephrotoxic agents is common, increasing in incidence and associated with considerable morbidity and mortality in developing countries. MicroRNAs are stable biomarkers that can be detected in extracellular fluids. This systematic scoping review aims to describe published research on urinary and circulating microRNAs in toxic acute kidney injury in both animal and human studies. We conducted a literature search, using EMBASE and Medline, for articles on urinary and circulating microRNA in nephrotoxic injuries to February 2020. A total of 21 publications studied acute kidney injury from 12 different toxic agents. Cisplatin was the most common nephrotoxic agent (n = 10), followed by antibiotics (n = 4). There were no randomized controlled trials. An increase in urinary miR‐218 predicted acute kidney injury in six different studies, suggesting it is a promising biomarker for nephrotoxin‐induced acute kidney injury. There were many factors that prevented a more comprehensive synthesis of microRNA performance including highly variable models, no consistent protocols for RNA isolation, cDNA synthesis and PCR amplification, and variability in normalization methods using reference controls. In conclusion, while microRNAs are promising biomarkers to study nephrotoxic acute kidney injury, the replication of most positive findings is not assessable due to deficient reporting of negative outcomes. A very narrow range of poisons have been studied, and more human data are required. In particular, further studies are needed on the most important causes of nephrotoxic injury, such as pesticides, chemicals, snake envenoming, and medicines other than aminoglycosides and cisplatin.

**Abbreviations**
AKI: Acute kidney injury
NAG: *N‐*acetyl‐β‐d‐glucosaminidase
SCr: serum creatinine

## 1 INTRODUCTION

Acute kidney injury (AKI) caused by drugs, chemicals, and toxins is a common clinical problem around the world. Deliberate self‐poisoning with pesticide and snake envenomation are typical causes of toxic AKI in developing countries,^1^ while toxic AKI from drugs such as chemotherapeutic agents is most common in western countries.^2^ A major challenge in the management of AKI is the only modest sensitivity of current injury biomarkers for early diagnosis.^3^, ^4^ These biomarkers have had limited translation into clinical medicine ^5^ and there is still an ongoing search for more sensitive and specific biomarkers for diagnosis of AKI. Better biomarkers of toxic AKI may help in earlier diagnosis, allowing early and appropriate treatment, and a means to monitor treatment effects.

MicroRNAs (miRNAs) are a class of RNAs that are potential diagnostic and prognostic biomarkers. MicroRNAs are endogenous, noncoding RNAs, 21‐25 nucleotides in size that regulate gene expression by binding to target messenger RNAs (mRNAs).^6^, ^7^, ^8^ They have potential advantages over protein biomarkers due to higher stability and tissue specificity, and the ease of quantifying large numbers of them simultaneously.^9^ Even though miRNAs were discovered in 1993, publications on miRNAs and kidneys only started from 2007, with rapidly growing interest in the last decade (Figure S1). Toxic agents lead to many changes in tissue miRNA expression. This may be detected in miRNA released into the systemic circulation, including when there is cell/tissue lysis due to injury. MicroRNAs can be detected in a range of biological samples, such as blood, urine, cerebrospinal fluid, feces, and breast milk.^10^ The majority of the reviews published so far focus on noncoding RNAs in various types of AKI and the information on miRNAs in nephrotoxic AKI is not organized. There are recent reviews published on noncoding RNAs in other types of AKI, but the limited information on miRNAs due to nephrotoxic AKI is not separable.^11^ Many studies report tissue miRNA regulation in nephrotoxic AKI. However, no systematic reviews have focused on miRNAs that might be easily and repeatedly measured in urine or blood as biomarkers of renal toxicity.

This systematic scoping review aims to describe published research on urinary and circulating miRNAs in nephrotoxic AKI in both animal and human studies. This will provide a useful basis for future studies that address cell‐free miRNA as biomarkers for toxin‐induced AKI.

## 2 MATERIALS AND METHODS

### 2.1 Search strategy

We conducted literature searches using EMBASE (1993 to February 2020) and Medline (1993 to February 2020) for articles on circulating miRNA in toxic AKI. We used a combination of sets of search terms: [(acute toxicity OR nephrotoxicity OR kidney injury OR kidney damage OR organ toxicity OR acute kidney injury OR toxin OR snakebite OR snake bite envenomation) AND (serum microRNA OR urinary microRNA OR plasma microRNA OR circulating microRNA OR cell‐free microRNA OR microRNA OR serum miRNA OR plasma miRNA OR Urinary miRNA OR circulating miRNA OR miRNA)]. We used Covidence systematic review management system for the title/abstract and full‐text screening. We restricted the search to the English language.

### 2.2 Screening for study relevance and eligibility

Randomized/nonrandomized trials, controlled/noncontrolled trials, cohort studies, and case‐control studies that investigated the use of miRNAs as biomarkers for the diagnosis or risk stratification of AKI following nephrotoxic agents (drugs and chemicals) were considered eligible. Furthermore, we included studies reporting on the detection of miRNAs in either urine or blood samples and individual miRNAs or global miRNA profiles in animal or human subjects. Two reviewers (FS and M.B) independently assessed the eligibility of the screened title and abstracts. Three reviewers (FS, F.M, and MB) independently screened full text for eligibility. N.B resolved any conflicts raised in the full‐text screening. We excluded studies if one of the following existed: (i) studies on tissue or cells, (ii) other types of induced AKI (eg, critical care, ischemia‐reperfusion injury, sepsis, posttransplant, and immune), (iii) studies with no available full text (eg, conference abstracts).

### 2.3 Data extraction

We extracted data into a prespecified data collection form to capture studied agents, experimental method, animal model or human study results, time and dose responses, and the number of miRNAs profiled (Table S1). We extracted data on the miRNAs identified within the site of injury and their expression in urine and serum.

## 3 RESULTS

### 3.1 Literature selection and study characteristics

**Figure 1:**
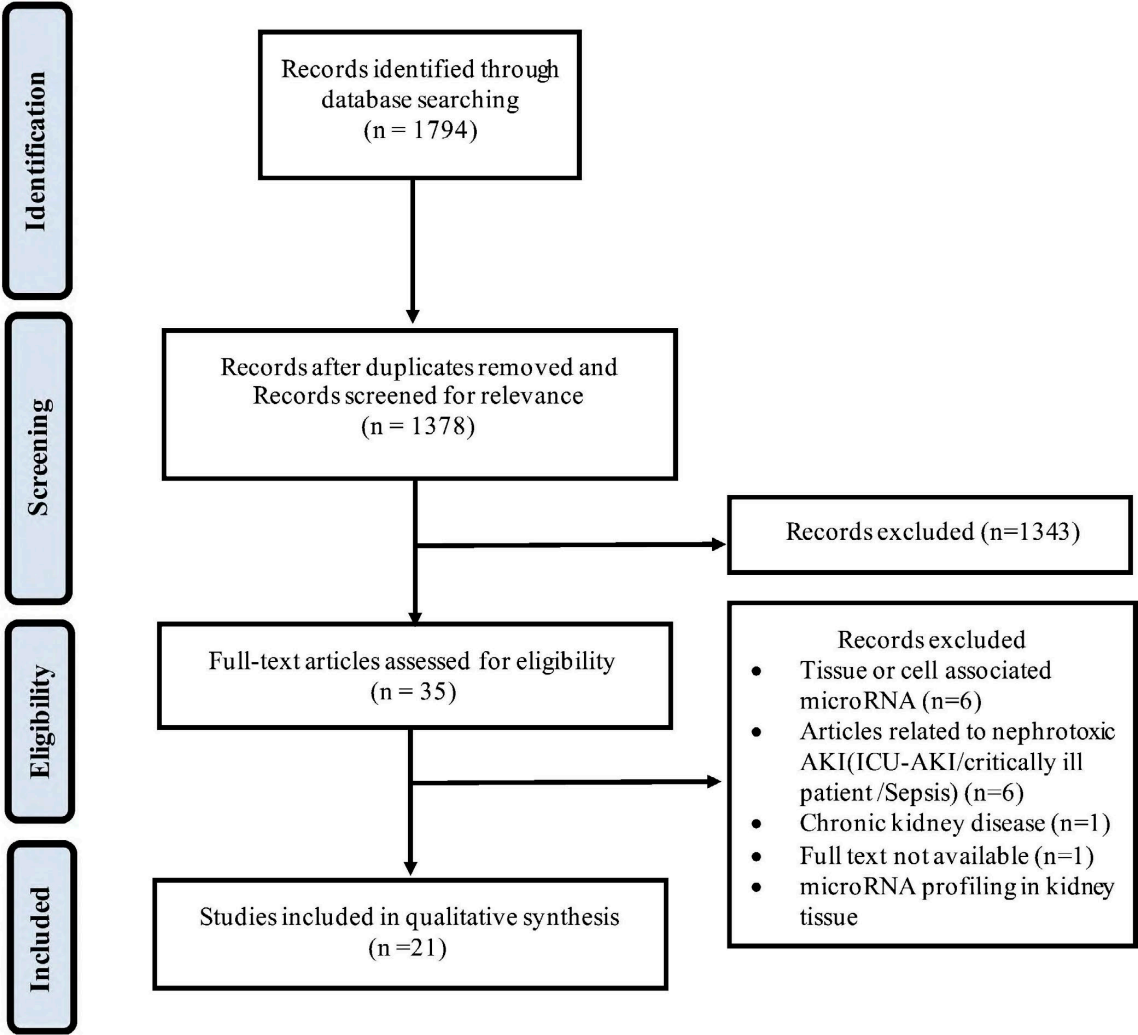
Search strategy flow diagram. The PRISMA diagram shows the process of literature identification, screening, eligibility, and study included in the review

Our search identified 1378 unique peer‐reviewed journal articles published since 2007. We then performed a detailed evaluation of each title and abstract and 1343 were excluded due to being other forms of AKI, miRNA studied in cells and tissues, review articles, conference abstracts or editorial/commentary, and book chapters. Finally, 35 articles were eligible for full‐text screening and we selected 21 for final analysis.^12^, ^13^, ^14^, ^15^, ^16^, ^17^, ^18^, ^19^, ^20^, ^21^, ^22^, ^23^, ^24^, ^25^, ^26^, ^27^, ^28^, ^29^, ^30^, ^31^, ^32^ The application of the screening process and selection criteria is summarized in Figure 1.

### 3.2 Nephrotoxic agents studied

There have been no studies on circulating or urinary miRNA in AKI induced by pesticides, chemicals, or snakebite envenoming. Studies to date have focused on chemotherapeutic agents such as cisplatin,^13^, ^14^, ^15^, ^16^, ^30^ adriamycin/doxorubicin,^13^, ^26^, ^31^ and antibiotics (gentamicin/puromycin ^13^, ^15^, ^20^, ^25^, ^29^, ^32^) (Table 1). Cisplatin,^23^ paracetamol,^23^, ^24^ cadmium,^12^ and contrast medium ^21^, ^27^ are the only agents studied in humans (Table 1).

**Table 1:** Number of articles published in human and animal model in nephrotoxic agents

Nephrotoxic agent	Human (n)	Animal (n)
Cisplatin	1	9
Gentamicin	‐	4
Contrast medium	2	2
Doxorubicin	‐	2
Aristolochic Acid I	‐	1
Adriamycin	‐	1
Cyanuric acid	‐	1
Melamine	‐	1
N‐phenylanthranylic acid	‐	1
Cadmium	1	‐
Paracetamol	2	‐
Puromycin	‐	3

Note

The numbers add up to more than 21 as three studies examined both humans and animals.^21^, ^24^, ^27^ and there were multiple agents studied in two publications.^13^, ^15^

### 3.3 miRNA profiling models and type of specimen

miRNA studies have been predominantly performed in animal models (16/21 and three studies in both animal and humans), usually rat models, (Table 1). All studies had limited power. Animal studies consistently had small numbers per group (ie n = 5 to 10); human studies ranged from 24 to 92 patients per group (Supplementary Table 2). The two largest studies were both on contrast medium‐induced AKI in humans.^21^, ^27^

**Table 2:** Human MicroRNAs profiling in nephrotoxic AKI

MicroRNA	Model	Expression in Urine	Expression in Serum	References
miR‐19a, miR‐19b	Paracetamol		↓	^24^
miR‐21	Cisplatin/Paracetamol/ Cadmium	↑	↑	12, 23
miR‐30a‐5p	Contrast medium		↑	21, 27
miR‐30e‐5p	Contrast medium		↑	21, 27
miR‐30b	Contrast medium		↑	^27^
miR‐34a‐5p	Paracetamol		↓	^24^
miR‐122‐5p	Paracetamol		NC	^24^
miR‐188‐5p	Contrast medium		↑	^21^
miR‐192‐5p	Paracetamol		↓	^24^
miR‐151‐3p	Paracetamol		NC	^24^
miR‐200c	Cisplatin/Paracetamol	↑		^23^
miR‐320	Contrast medium		↑	^27^
miR‐382‐5p	Paracetamol		NC	^24^
miR‐423	Cisplatin/Paracetamol	↑		^23^
miR‐885‐5p	Paracetamol		NC	^24^
miR‐3187‐3p	Paracetamol		↓	^24^

Up and down arrows and NC represent the up‐regulated, down‐regulated and no changes in miRNA expression.

Only two studies simultaneously examined miRNA expression in the circulation, urine and tissue in animal models.^16^, ^32^ One reported increases in two miRNA in kidney tissue with decreased levels in plasma and urine.^32^ The other only reported statistically significant changes in miRNAs, and reported minimal overlap in results from kidney, plasma, and urine.^16^ Rat studies generally used outbred stocks (Wistar and Sprague‐Dawley, 14/15) while mice studies more often used inbred strains (Balb/c, 2/4) (Table S2).

### 3.4 miRNA detection techniques

The majority of studies used TaqMan low‐density array (TLDA) card RT‐qPCR detection technique (9/21) or SYBR Green RT‐qPCR (5/21) (Table S2). Few studies profiled candidate miRNAs and then validated the best candidates in the same model.^16^, ^29^

### 3.5 Comparison of miRNA profiling in animal model and humans

Two studies on contrast medium‐induced AKI showed higher expression of serum miR‐30a‐5p, miR‐30e‐5p, and miR‐30b in both animal models and humans (Table 2).^21^, ^27^ There were no urine miRNAs that had comparable changes in both animals and humans, noting there was only one human study examining urinary miRNA.^23^

### 3.6 Confirmation of kidney injury and categorization of different stages of AKI

Most animal studies confirmed AKI by histopathological examination of kidney tissue alone (11/16) or histology with serum creatinine (SCr) (5/16). In contrast, human studies used SCr,^21^, ^24^, ^27^ *N‐*acetyl‐β‐d‐glucosaminidase (NAG),^12^ or histopathology ^23^ to confirm AKI (Table S2). Grading of severity of AKI was generally done in animal studies but not in human studies.

### 3.7 miRNA in tubular and glomerular injury

**Figure 2:**
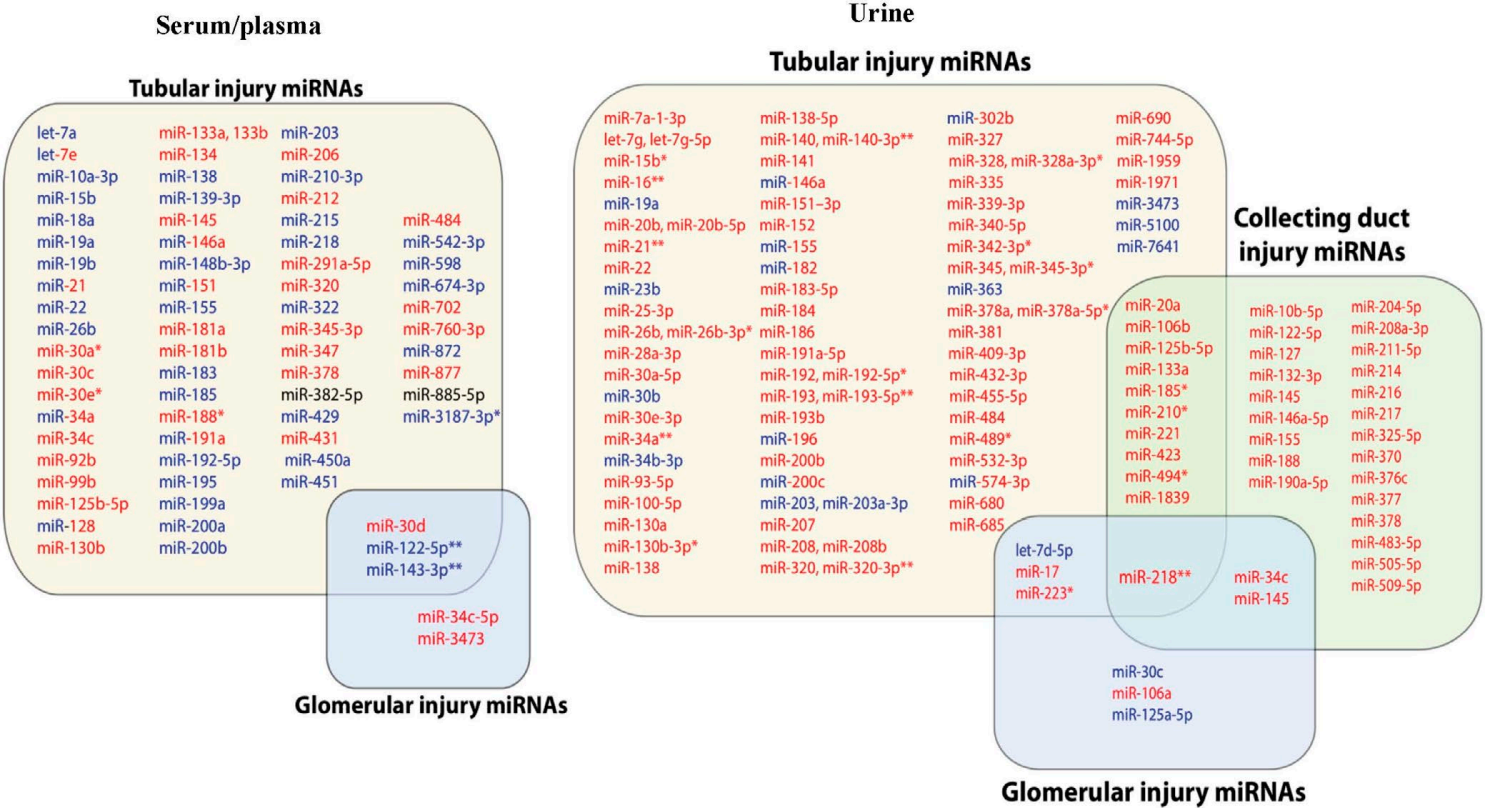
Expression of serum/urine and urinary miRNAs at different sites of injury after nephrotoxin exposure in either animal model or humans. Overexpressed, underexpressed, and unchanged miRNAs are highlighted in red, blue, and black in colors, respectively. miRNAs in red and blue colors together show miRNAs that had conflicting results in different studies. * ‐ miRNA identified in two different studies. ** ‐ miRNA identified in three or more studies

Tubular injury models were most commonly studied. We identified a total of 110 urinary and 72 circulating miRNAs associated with nephrotoxic AKI in at least one study. There were 20 specific miRNAs that were associated in both urine and circulation. Toxic AKI generally led to elevated miRNAs in urine ^14^, ^33^ and downregulated circulating miRNAs.^12^, ^27^, ^32^ Elevated urinary miR‐218 predicted AKI in six different studies, suggesting it is a promising biomarker for nephrotoxin‐induced AKI. Two circulating miRNAs (miR‐122‐5p and miR‐143‐3p) were downregulated in toxic AKI in three separate studies (Figure 2). There were four miRNAs (miR‐17, miR‐106a, miR‐218 and miR‐223) that increased in urine after both glomerular and tubular injury (Figure 2 ).

### 3.8 Normalization of serum and urinary miRNA levels

All studies normalized miRNAs to a spike‐in control miRNA (specifically, serum‐reference genes: U6/cel‐miR‐39/miR‐1287/miR‐342‐3p/ath‐miR‐159a and urine‐reference genes: ath‐miR‐159a/U6/let‐7d‐5p, miR‐16‐5p, miR‐191‐5p/5s ribosomal RNA). In six of eleven urinary miRNA studies, these normalized cycle threshold (Ct) values were not adjusted using urinary creatinine (UCr), while other publications used three different methods of adjusting miRNA expression using UCr (Table S2).

#### 3.8.1 Quality measures in the selected studies

**Figure 3:**
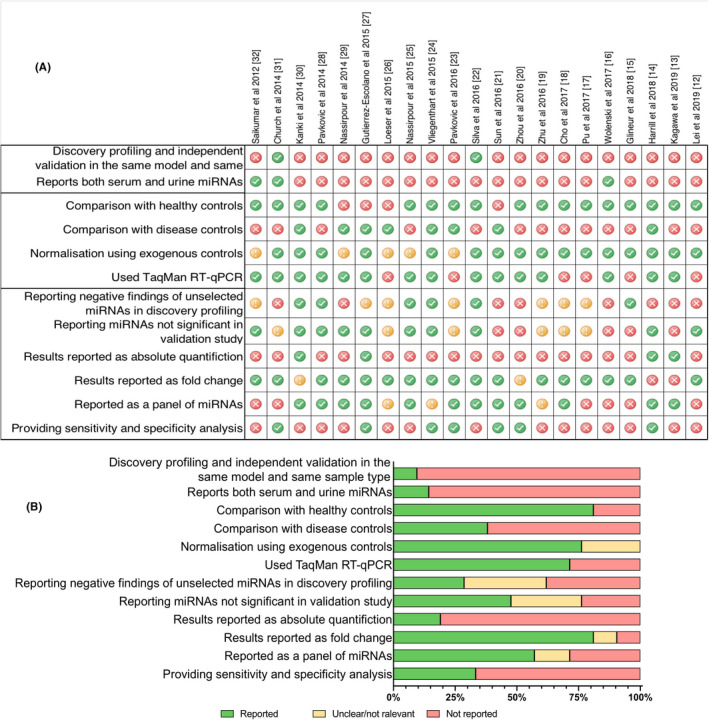
Quality measures included in 21 studies (A) Summary as heat map and (B) percentage. Quality of study determined by the authors. Green, light brown, and red indicate reported, unclear/not relevant, and not reported, respectively

We systematically analyzed the studies further to assess the quality of the study in terms of study design, the methods, and the outcome (Figure 3A and B).

##### Study design

We found only two studies performed global profiling and validated the selected miRNAs in an independent study sample using the same model and same sample type.^22^, ^31^ Only three studies examined miRNA expression in both circulation and urine.^16^, ^31^, ^32^

##### Method

The majority of studies used healthy controls (ie, sham group in animal models) while only three studies also used disease controls (poisoned but no kidney injury).^15^, ^26^, ^29^ TaqMan probe‐based RT‐qPCR technique was widely used with exogenous controls for normalization.

##### Study outcome

In general, the studies focused reporting on positive results. Only six studies provided negative findings as a list or as a heat map from global profiling, and only half of the studies reported miRNAs with nonsignificant results in the validation study. Most studies presented the results as fold change compared to control (80%) while less than 20% of the studies quantified absolute CT values. Only 34% of the studies analyzed receiver operating characteristics or sensitivity/specificity of significantly associated miRNAs.

## 4 DISCUSSION

This review identified only 21 studies on circulating or urinary miRNAs in nephrotoxic AKI in animal models and humans, mainly concerning drug‐induced AKI. We found no studies that investigated important nondrug agents such as pesticides, chemicals, and envenoming induced AKI.

There were even fewer human studies investigating miRNAs in toxic AKI. All studies selected miRNAs that had previously been investigated, mostly from prior rat models or from literature. Only two studies examined global miRNA profile which would allow identification of potential novel miRNA biomarkers and validated in the same sample type and model. Only one study compared miRNA expression to clinically relevant control groups without AKI as well as healthy controls.^23^ Global profiling conducted in discovery cohorts and validated in independent large sets of cohorts, and comparisons with other patients as well as healthy controls,^34^ are required to identify the most promising miRNA biomarkers. There were only three animal studies investigating miRNAs in toxic AKI using inbred strains. The use of inbred strains for toxicological studies is often suggested as they are more stable, better defined, more uniform, and provide more repeatable data than outbred stocks.^35^, ^36^ Complete inbreeding is an unusual genetic state for a mammal that does not accurately reflect the human genetic makeup. Therefore, the use of Diversity Outbred mice, a newly developed mouse population derived from progenitor lines of the Collaborative Cross is highly recommended as a powerful model system that more closely reflects the genetic variability seen in humans.^37^ Advantages of Diversity Outbred mice in genetics are higher mapping resolution, increased heterozygosity, and uniformly distributed genetic variation across the genome.^38^

Knowledge of the specificity of the miRNA is also required to define the clinical utility of potential biomarkers. The change in miRNA expression might be specific to one or a wide range of toxic agents, they may or may not be species specific. We found only a few miRNAs that were shown multiple times to detect AKI after nephrotoxic agent exposure. Notably, miR‐218 was associated with cisplatin, gentamicin, and puromycin‐induced AKI.^14^, ^15^, ^20^, ^25^, ^29^, ^30^ MiR‐218 regulates cell migration via the Slit‐Robo pathway and promotes the motility of renal tubular epithelial cells that undergo epithelial mesenchymal transition.^39^ Thus upregulation of miR‐218 might indicate tubular AKI and cell apoptosis from any tubular nephrotoxic process.^40^ In contrast, some miRNA may be specific to a particular toxic injury.^41^

Rat models were generally the models used for in vivo animal studies. However, different miRNA profiles were often reported for the same nephrotoxic agent. For example, three cisplatin rat models did not identify any common urinary miRNAs after AKI.^16^, ^28^, ^30^ This concerning discrepancy in reported miRNA expression was not explored by the authors, but might feasibly be due to variable rat strains, doses, sample collection times, or different techniques used in each study. Even greater discrepancies would be expected between animal and human studies.^42^

Useful new clinical diagnostic biomarkers need to detect AKI earlier than the traditional biomarker SCr, ideally with no additional assays, adjustments, or calculations. Interestingly, adjusting for age and gender did not change the diagnostic performance of miR‐21 and miR‐423 in paracetamol‐induced AKI.^23^ UCr is often used to normalize for urine dilution in assays of urinary protein biomarkers. Results adjusted for urine dilution were presented by roughly half the studies. However, normalizing miRNA to UCr or urine volume may be superfluous as typically the miRNA concentrations were elevated around a million‐fold. Ideally studies would report both absolute and normalized values for urinary protein biomarkers,^43^ and demonstrate if this was unnecessary; however, this was not done by any studies.

### 4.1 Limitations of the review

One limitation of this systematic review is that we only included articles in English; however, we did not identify any relevant non‐English articles with our search terms. Additionally, we did not perform any meta‐analysis on the specificity and sensitivity of miRNAs due to extreme heterogeneity in the 21 studies, including differences across miRNAs, study models, types of agents, types of samples, and different qPCR techniques. Variation in normalization methods and presentation of results (fold change or fold over detectable or log transform) also were major barriers to statistical synthesis of biomarker performance.

### 4.2 A check list for complete reporting for future studies to facilitate meta‐analysis

#### 4.2.1 Study Design

Perform global profiling and validate the results in independent samples.

Profile and validate using the same model and sample type (serum or urine).

Use both disease controls and healthy controls as well as the kidney injury group.

#### 4.2.2 Method

Normalize using combinations of control miRNAs (exogenous and endogenous).

Validate the results using the same or secondary technique such as real time or digital PCR.

### 4.3 Outcome

Compare the results with healthy controls and disease controls.

Report both absolute quantification and the fold change.

Provide sensitivity and specificity analysis (ROC‐AUC).

Report all analyzed miRNAs (significant and nonsignificant).

## 5 CONCLUSION AND FUTURE DIRECTIONS

While miRNA are promising biomarkers to study nephrotoxic AKI, we identified (i) lack of attempts to replicate most findings, (ii) no consistent protocols for RNA isolation, cDNA synthesis, and PCR amplification, (iii) variability in methods used to normalize to reference controls (iv) some major discrepancies between study findings (perhaps explained by i‐iii, above), (v) a very narrow range of poisons studied, and (vi) a dearth of human data. Thus, we require more studies in both humans and animals on other important causes of nephrotoxic injury, such as pesticides, chemicals, envenoming, and medicines other than aminoglycosides and cisplatin. Standardizing techniques and reference controls and/or reporting absolute quantification would allow for better comparisons between studies.

## 6 ETHICS APPROVAL STATEMENT

No ethical approval required for the systematic review.

## DISCLOSURES

The authors declare that they have no conflict of interest.

## AUTHOR CONTRIBUTIONS

Buckley, Seth, and Shihana participated in research design. Shihana, Mohamed, and Barron contributed the analytic tools. Shihana performed the data analysis. Shihana, Seth, and Buckley wrote or contributed to the writing of the manuscript.

## Supporting information

Table S1‐S2

## Data Availability

The data that support the findings of this study are available from the corresponding author upon reasonable request.

## References

[1] Mohamed F , Endre ZH , Buckley NA . Role of biomarkers of nephrotoxic acute kidney injury in deliberate poisoning and envenomation in less developed countries. Br J Clin Pharmacol. 2015;80(1):3‐19. 26099916 10.1111/bcp.12601 PMC4500319

[2] Pannu N , Nadim MK . An overview of drug‐induced acute kidney injury. Crit Care Med. 2008;36(4 Suppl):S216‐S223. 18382197 10.1097/CCM.0b013e318168e375

[3] Murray PT , Mehta RL , Shaw A , et al. Potential use of biomarkers in acute kidney injury: report and summary of recommendations from the 10th Acute Dialysis Quality Initiative consensus conference. Kidney Int. 2014;85(3):513‐521. 24107851 10.1038/ki.2013.374 PMC4198530

[4] Ho J , Tangri N , Komenda P , et al. Urinary, plasma, and serum biomarkers' utility for predicting acute kidney injury associated with cardiac surgery in adults: a meta‐analysis. Am J Kidney Dis. 2015;66(6):993‐1005. 26253993 10.1053/j.ajkd.2015.06.018

[5] Griffin BR , Gist KM , Faubel S . Current status of novel biomarkers for the diagnosis of acute kidney injury: a historical perspective. J Intensive Care Med. 2020;35(5):415‐424. 30654681 10.1177/0885066618824531 PMC7333543

[6] Ambros V . The functions of animal microRNAs. Nature. 2004;431(7006):350‐355. 15372042 10.1038/nature02871

[7] Bartel DP . MicroRNAs: genomics, biogenesis, mechanism, and function. Cell. 2004;116(2):281‐297. 14744438 10.1016/s0092-8674(04)00045-5

[8] Bartel DP . MicroRNAs: target recognition and regulatory functions. Cell. 2009;136(2):215‐233. 19167326 10.1016/j.cell.2009.01.002 PMC3794896

[9] Huang W . MicroRNAs: Biomarkers, diagnostics, and therapeutics. Methods Mol Biol. 2017;1617:57‐67. 28540676 10.1007/978-1-4939-7046-9_4

[10] Weber JA , Baxter DH , Zhang S , et al. The microRNA spectrum in 12 body fluids. Clin Chem. 2010;56(11):1733‐1741. 20847327 10.1373/clinchem.2010.147405 PMC4846276

[11] Fan PC , Chen CC , Chen YC , Chang YS , Chu PH . MicroRNAs in acute kidney injury. Hum Genomics. 2016;10(1):29. 27608623 10.1186/s40246-016-0085-z PMC5016954

[12] Lei LJ , Zhang Z , Guo JY , et al. MiR‐21 as a potential biomarker for renal dysfunction induced by cadmium exposure. Int J Clin Exper Med. 2019;12(2):1631‐1639.

[13] Kagawa T , Zarybnicky T , Omi T , et al. A scrutiny of circulating microRNA biomarkers for drug‐induced tubular and glomerular injury in rats. Toxicology. 2019;415:26‐36. 30682439 10.1016/j.tox.2019.01.011

[14] Harrill AH , Lin H , Tobacyk J , Seely JC . Mouse population‐based evaluation of urinary protein and miRNA biomarker performance associated with cisplatin renal injury. Exp Biol Med (Maywood). 2018;243(3):237‐247. 29110506 10.1177/1535370217740854 PMC5813868

[15] Glineur SF , Hanon E , Dremier S , et al. Assessment of a urinary kidney MicroRNA panel as potential nephron segment‐specific biomarkers of subacute renal toxicity in preclinical rat models. Toxicol Sci 2018;166(2):409‐419. 30169741 10.1093/toxsci/kfy213

[16] Wolenski FS , Shah P , Sano T , et al. Identification of microRNA biomarker candidates in urine and plasma from rats with kidney or liver damage. J Appl Toxicol. 2017;37(3):278‐286. 27397436 10.1002/jat.3358 PMC5298042

[17] Pu XY , Shen JY , Deng ZP , Zhang ZA . Plasma‐specific microRNA response induced by acute exposure to aristolochic acid I in rats. Arch Toxicol. 2017;91(3):1473‐1483. 27422293 10.1007/s00204-016-1791-y

[18] Cho YE , Kim SH , Lee BH , Baek MC . Circulating plasma and exosomal microRNAs as indicators of drug‐induced organ injury in rodent models. Biomolecules and Therapeutics. 2017;25(4):367‐373. 28208010 10.4062/biomolther.2016.174 PMC5499614

[19] Zhu Y , Yu J , Yin L , et al. Microrna‐146b, a sensitive indicator of mesenchymal stem cell repair of acute renal injury. Stem Cells Transl Med. 2016;5(10):1406‐1415. 27400799 10.5966/sctm.2015-0355 PMC5031179

[20] Zhou X , Qu Z , Zhu C , et al. Identification of urinary microRNA biomarkers for detection of gentamicin‐induced acute kidney injury in rats. Regul Toxicol Pharmacol. 2016;78:78‐84. 27074385 10.1016/j.yrtph.2016.04.001

[21] Sun SQ , Zhang T , Ding D , et al. Circulating MicroRNA‐188, ‐30a, and ‐30e as early biomarkers for contrast‐induced acute kidney injury. J Am Heart Assoc. 2016;5(8);(no pagination)(e004138). 10.1161/JAHA.116.004138 PMC5015315 27528406

[22] Silva CS , Chang CW , Williams D , Porter‐Gill P , Gamboa da Costa G , Camacho L . Effects of a 28‐day dietary co‐exposure to melamine and cyanuric acid on the levels of serum microRNAs in male and female Fisher 344 rats. Food Chemical Toxicol 2016;Part A. 98:11‐16. 10.1016/j.fct.2016.09.013 PMC5086269 27621052

[23] Pavkovic M , Robinson‐Cohen C , Chua AS , et al. Detection of Drug‐Induced Acute Kidney Injury in Humans Using Urinary KIM‐1, miR‐21, ‐200c, and ‐423. Toxicol Sci. 2016;152(1):205‐213. 27122240 10.1093/toxsci/kfw077 PMC5009468

[24] Vliegenthart AD , Shaffer JM , Clarke JI , et al. Comprehensive microRNA profiling in acetaminophen toxicity identifies novel circulating biomarkers for human liver and kidney injury. Sci Rep. 2015;5:15501. 26489516 10.1038/srep15501 PMC4614545

[25] Nassirpour R , Homer BL , Mathur S , et al. Identification of Promising Urinary MicroRNA Biomarkers in Two Rat Models of Glomerular Injury. Toxicol Sci. 2015;148(1):35‐47. 26253709 10.1093/toxsci/kfv167

[26] Loeser H , von Brandenstein M , Herschung A , Schlosser M , Buttner R , Fries JW . ET‐1 Induced Downregulation of MRP2 via miRNA 133a ‐ A Marker for Tubular Nephrotoxicity? Am J Nephrol. 2015;41(3):191‐199. 25871823 10.1159/000381272

[27] Gutierrez‐Escolano A , Santacruz‐Vazquez E , Gomez‐Perez F . Dysregulated microRNAs involved in contrast‐induced acute kidney injury in rat and human. Ren Fail. 2015;37(9):1498‐1506. 26337190 10.3109/0886022X.2015.1077322

[28] Pavkovic M , Riefke B , Ellinger‐Ziegelbauer H . Urinary microRNA profiling for identification of biomarkers after cisplatin‐induced kidney injury. Toxicology. 2014;324:147‐157. 24880025 10.1016/j.tox.2014.05.005

[29] Nassirpour R , Mathur S , Gosink MM , et al. Identification of tubular injury microRNA biomarkers in urine: comparison of next‐generation sequencing and qPCR‐based profiling platforms. BMC Genom. 2014;15:485. 10.1186/1471-2164-15-485 PMC4079956 24942259

[30] Kanki M , Moriguchi A , Sasaki D , et al. Identification of urinary miRNA biomarkers for detecting cisplatin‐induced proximal tubular injury in rats. Toxicology. 2014;324:158‐168. 24863737 10.1016/j.tox.2014.05.004

[31] Church RJ , McDuffie JE , Sonee M , et al. MicroRNA‐34c‐3p is an early predictive biomarker for doxorubicin‐induced glomerular injury progression in male Sprague‐Dawley rats. Toxicol. Res. 2014;3(5):384‐394.

[32] Saikumar J , Hoffmann D , Kim TM , et al. Expression, circulation, and excretion profile of microRNA‐21, ‐155, and ‐18a following acute kidney injury. Toxicol Sci. 2012;129(2):256‐267. 22705808 10.1093/toxsci/kfs210 PMC3499041

[33] Kito N , Endo K , Ikesue M , Weng H , Iwai N . miRNA Profiles of Tubular Cells: Diagnosis of Kidney Injury. Biomed Res Int. 2015;2015:465479. 26106607 10.1155/2015/465479 PMC4461729

[34] Shihana F , Joglekar MV , Raubenheimer J , Hardikar AA , Buckley NA , Seth D . Circulating human microRNA biomarkers of oxalic acid‐induced acute kidney injury. Arch Toxicol. 2020. 10.1007/s00204-020-02679-5 32086547

[35] Festing MF . Inbred strains should replace outbred stocks in toxicology, safety testing, and drug development. Toxicol Pathol. 2010;38(5):681‐690. 20562325 10.1177/0192623310373776

[36] Festing MF . Genetically Defined Strains in Drug Development and Toxicity Testing. Methods Mol Biol. 2016;1438:1‐17. 27150081 10.1007/978-1-4939-3661-8_1

[37] Svenson KL , Gatti DM , Valdar W , et al. High‐resolution genetic mapping using the Mouse Diversity outbred population. Genetics. 2012;190(2):437‐447. 22345611 10.1534/genetics.111.132597 PMC3276626

[38] Smallwood TL , Gatti DM , Quizon P , et al. High‐resolution genetic mapping in the diversity outbred mouse population identifies Apobec1 as a candidate gene for atherosclerosis. G3: Genes ‐ Genomes ‐ Genetics. 2014;4(12):2353‐2363. 25344410 10.1534/g3.114.014704 PMC4267931

[39] Zarjou A , Yang S , Abraham E , Agarwal A , Liu G . Identification of a microRNA signature in renal fibrosis: role of miR‐21. Am J Physiol Renal Physiol. 2011;301(4):F793‐801. 21775484 10.1152/ajprenal.00273.2011 PMC3191802

[40] Zhang T , Xiang L . Honokiol alleviates sepsis‐induced acute kidney injury in mice by targeting the miR‐218‐5p/heme oxygenase‐1 signaling pathway. Cell Mol Biol Lett. 2019;24:15. 30833971 10.1186/s11658-019-0142-4 PMC6387556

[41] Kato M , Park JT , Natarajan R . MicroRNAs and the glomerulus. Exp Cell Res. 2012;318(9):993‐1000. 22421514 10.1016/j.yexcr.2012.02.034 PMC3334455

[42] Barnett LMA , Cummings BS . Nephrotoxicity and renal pathophysiology: a contemporary perspective. Toxicol Sci. 2018;164(2):379‐390. 29939355 10.1093/toxsci/kfy159

[43] Tang KW , Toh QC , Teo BW . Normalisation of urinary biomarkers to creatinine for clinical practice and research–when and why. Singapore Med J. 2015;56(1):7‐10. 25640093 10.11622/smedj.2015003 PMC4325562

