# Supplementary material for: MicroRNAs in toxic acute kidney injury: Systematic scoping review of the current status
Source: Pharmacol Res Perspect. 2021 Feb 18;9(2):e00695. doi: 10.1002/prp2.695 (PMC7891060; doi:10.1002/prp2.695)
Supplement: Supplementary file 1 — Table S1‐S2 [file PRP2-9-e00695-s001.docx]

**Title: MicroRNAs in toxic acute kidney injury: Systematic scoping review of the current status**

Fathima Shihana M.Phil^1,2^

Melissa L. Barron PhD^2^

Fahim Mohamed PhD^1,2,3^

Devanshi Seth PhD ^4-6^

Nicholas A Buckley MD ^1,2^

^1^Clinical Pharmacology and Toxicology Research Group, Discipline of Pharmacology, Faculty of Medicine and Health, The University of Sydney, Sydney, NSW, Australia.

^2^South Asian Clinical Toxicology of Research Collaboration, Faculty of Medicine, University of Peradeniya, Sri Lanka.

^3^Allied Health Sciences, Department of Pharmacy, University of Peradeniya, Sri Lanka.

^4^Discipline of Clinical Medicine & Addiction Medicine, Faculty of Medicine and Health, The University of Sydney, NSW, Australia.

^5^Drug Health Services, Royal Prince Alfred Hospital, Camperdown, NSW, Australia.

^6^The Centenary Institute of Cancer Medicine & Cell Biology, The University of Sydney, NSW, Australia.

Molecular Pharmacology

MOLPHARM-AR-2020-000137

Supplementary Table1: Criteria used for the data extraction

| Heading | Data extraction criteria |
| --- | --- |
| Nephrotoxic agents | Chemotherapeutic drugs, antibiotics, pesticides, Plant toxins, Snake envenomation |
| Study model | Human, Rat, Mouse |
| miRNA detection method | TaqMan Probe, SYBR Green, micro Array, miRNA sequencing |
| Dose | amount of toxin used to induce AKI |
| Sampling point | Time to collect samples for MIRNA assay |
| Type of Sample | Serum, Plasma, Urine |
| Number of MicroRNA Profiled | Single , Multiple |
| Differentially expressed miRNA | Upregulated, down regulated, unchanged |
| Correlation with any other functional or injury biomarker | SCr, NAG, KIM-1 |
| Site of injury | Proximal tubular injury, Glomerular injury, collecting duct |

Supplementary Table 2: Evaluation of miRNA as biomarkers in AKI

| **Nephrotoxic Agents** | **Species**  **(strain)** | **Sample type/number** | **Sampling points/** | | **AKI confirmation** | **Number of miRNA Profiled** | **miRNA assay technique** | **Regulation of miRNA** | **Correlation** | **Normalisation of urinary miRNA (adjusting to UCr)** |
| --- | --- | --- | --- | --- | --- | --- | --- | --- | --- | --- |
| Adriamycin[26] | Mouse  (Balb/c) | Urine  N=5:5 | Day 14, 28 | Proteinuria | | 1 | Sanger Sequencing | miRNA 133a 🡱 | Not studied | Not adjusted |
| Aristolochic  Acid I[17] | Rat  (Wistar) | Plasma  N=5:5 | Day 2, day 4, and day 6 | Histopathology | | 1 | Agilent microarray assay | miR-21-3p 🡱 | Not studied | Not applicable |
| Cadmium[12] | Human | Plasma  N=31:24 | NA | Kidney dysfunction (NAG) | | 2 | SYBR Green- RT-qPCR | miR-21🡱 | miR-21was significantly correlated with normalised uNAG and UCd | Not applicable |
| Cisplatin[14] | Mouse  (DO) | Urine  N=44:45 | 18 h | Histopathology | | 335 | TaqMan® Low Density Array Cards | miR-130a, miR-151-3p, miR-320, miR-680, miR-152, miR-221,  miR-328, miR-218, miR-138,  miR-685 🡱 | Not studied | converting △Ct to linear scale (2^-△Ct^)/urine volume |
| Cisplatin[16] | Rat  (Sprague–Dawley) | Urine  N=4:4 | Day 1, 3 | Histopathology | | 832 | miRNA seq | miR-387a, miR-1839, miR-140, miR-26b, miR-22, let-7g 🡱 | Not studied | Not adjusted |
| Cisplatin[16] | Rat  (Sprague–Dawley) | Plasma  N=4:4 | Day 1, 3 | Histopathology | | 832 | miRNA seq | miR-34c, miR128, miR-34a, miR130b, miR-702, miR-6215, miR-484, miR-134, miR-151, let-7e, miR-191a, miR-431, miR-181b, miR-92b 🡱 | Not studied | Not adjusted |
| Cisplatin[28] | Rat  (Wistar) | Urine  N=6:6 | Day 3, 5, 8, 15 and 26. | Histopathology | | 373 | GeneChip Rat genome | miR-15, miR-16, miR-20a, miR-192, miR-193 and miR-210 🡱 | Not studied | converting △Ct to linear scale detectable level 2^(30-Ct)^ and divided by UCr (2^(30-Ct)^/UCr) |
| Cisplatin[30] | Rat  (Sprague–Dawley) | Urine  N=10:10 | Day 5, 0-1, 2 - 3, and 6-7 | SCr/Histopathology | | 322 | TaqMan microRNA assays | let-7g-5p, miR-1959, miR-138, miR-17-5p, miR-183-5p, miR-1839-5p,  miR-191a-5p,  miR-193-5p, miR-26b-3p, miR-30a-5p, miR-320-3p,  miR-328a-3p,  miR-93-5p, miR-532-3p,  miR-1274a, miR-744-5p, miR-7a-1-3p, let-7a-1-3p, miR-130b-3p, miR-140-3p, miR-192-5p, miR-196b-5p, miR-196c-5p, miR-20b-5p, miR-218a-5p, miR-25-3p, miR-335, miR-340-5p, miR-378a-5p,  miR-7a-1-3p 🡱 | Let-7g-5p, miR-93-5p, miR-191a-5p and miR-192-5p had positive correlation with BNU, SCr, KIM-1 and Clusterin | converting △Ct to linear 2^(△Ct)^ and multiplied by the fixed value (1000) and urine volume (ml) |
| Cisplatin[23] | Human | Urine  N=65:27 | 4, 8, 12, 24, 48, 72, 96, 120, and 144 h. | Histopathology | | 3 | SYBR Green- RT-qPCR | miR-21, miR-200c, miR-423 🡱 | miR-21, miR-200c, miR-423 weakly correlated with KIM-1 and SCr | converting △Ct to linear scale detectable level 2^(30-Ct)^ and divided by UCr (2 ^(30-Ct)^/UCr) |
| Cisplatin[13] | Rat  (Sprague–Dawley) | Plasma  N=5:5 | Day 1, 2, 3 and 5 | Histopathology | | 372 | Illumina MiSeq platform/TaqMan microRNA assays | miR-143-3p miR-122 was 🡳 | Not studied | Not applicable |
| Cisplatin[19] | Rat  (SpragueDawley) | Serum  N=6:6 | Day 1, 2, 3, 4, and 5 | Histopathology | | 1 | SYBR Green- RT-qPCR | miR-146b 🡱 | Not studied | Not applicable |
| Cisplatin[18] | Mouse  (Balb/C) | Plasma  N=5:5 | Day 1 | Histopathology | | 1 | SYBR Green- RT-qPCR | miR-146a🡱 | Not studied | Not applicable |
| Cisplatin[24] | Mouse  (CD1) | Plasma  N/A | Day 3 | KIM-1/  Histopathology | | 4 | SYBR Green PCR Kit/miScript miRNA QC PCR Array | miR-151-3p 🡳 | Not studied | Not applicable |
| Cisplatin[15] | Rat  (Wistar) | Urine  N=6:6 | 0, Day 1, 3, 6, 13, 20 and 27 | Histopathology | | 68 | FirePlex miRNA assay | miR-34c-5p 🡱 | miR-34c-5p was significantly correlated with KIM-1 | Not adjusted |
| Contrast medium[21] | Rat  (Sprague–Dawley) | Plasma  N/A | 8 hours | SCr/  Histopathology | | 350 | Agilent microarray | miR-347, miR-188, let-7b, miR-466b, miR-335, miR-30b-3p, miR-330, miR-483, miR-494, miR-30a, miR-760-5p, miR-877, miR-291a-5p, miR-30d, miR-181a, miR-30e, miR-345-3p, miR-296, miR-99b, miR-378, miR-378,  miR-125b-5p 🡱  miRNAs miR-342-5p,  miR-7a, miR-205 miR-34c, miR-542-5p, miR-215, miR-322, miR-203, miR-598-5p, miR-200a, miR-98, miR-429, miR-28, miR-183, miR-450a, miR-151, miR-15b, miR-10a-3p, let-7e, miR-126, miR-7a, miR-148b-3p, miR-872, miR-26b, miR-542-3p, miR-451, miR-199a-3p, miR-146b, miR-195, miR-204, miR-674-3p, miR-200b, miR-138, miR-139-5p, miR-363, miR-139-3p,  miR-185🡳 | Not studied | Not applicable |
| Contrast medium[21] | Human | Plasma  N=71:71 | 4 hours | SCr/  Histopathology | | 3 | TaqMan microRNA assays | miR-188-5p, miR-30a-5p,  miR-30e-5p 🡱 | miR-188-5p, miR-30a-5p, miR-30e-5p \associated with eGFR and correlated with SCr and CysC | Not applicable |
| Contrast medium[27] | Rat  (Sprague–Dawley) | Plasma  N/A | 24 hours | SCr and creatinine clearance | | 17 | TaqMan microRNA assays | miR-30a, miR-30 b,miR-30e and, miR-320 🡱  let-7a and miR-200a 🡳 | Not studied | Not applicable |
| Contrast medium[27] | Human | Plasma  N=92:92 | 24 hours | SCr and creatinine clearance | | 3 | TaqMan microRNA assays | miR-30a, miR-30c,miR-30e 🡱 | Peak miR-30a and miR-30e were correlated with peak SCr | Not applicable |
| Cyanuric acid[22] | Rat  (F344 rats) | Serum  N=12:12 | Day 28 | SCr/  Histopathology | | 752 | Exiqon qPCR array/ TaqMan miRNA assays | miR-128-3p was miR-22-3p, miR-191a-3p, miR-210-3p 🡳 | Not studied | Not applicable |
| Doxorubicin[31] | Rat  (Sprague–Dawley) | Urine  N=6:6 | Day 2, 7, 10, and 14 | SCr | | 750 | TaqMan Rodent miRNA arrays | miR-34c-3p 🡱 | miR-34c-3p correlated with urinary albumin | Not applicable |
| Doxorubicin[31] | Rat  (Sprague–Dawley) | Plasma  N=6:6 | Day 2, 7, 10, and 14 | SCr/ Histopathology | | 750 | TaqMan Rodent miRNA arrays | miR-34c-3p 🡱 | miR-34c-3p correlated with urinary albumin | Not applicable |
| Doxorubicin[13] | Rat  (Sprague–Dawley) | Plasma  N=5:5 | Day 3, 5, 7,10,14 and 21 | Histopathology | | 372 | Illumina MiSeq platform/TaqMan microRNA assays | miR-3473 🡱  miR-143-3p miR-122 🡳 | Not studied | Not applicable |
| Gentamicin[13] | Rat  (Sprague–Dawley) | Plasma  N=5:5 | Day 2, 3, 4, 5 and 7 | Histopathology | | 372 | Illumina MiSeq platform/TaqMan microRNA assays | miR-143-3p miR-122 🡳 | Not studied | Not applicable |
| Gentamicin[20] | Rat  (Wistar) | Urine  N=6:6 | Day 2, 4, 8, and 11 | Histopathology | | 370 | TaqMan® Low Density Array Cards | miR-539-3p, miR-690, miR-484, miR-140-3p, miR-342-3p, miR-138-5p, miR-16-3p, miR-345-5p, miR-28a-3p, miR-218-3p, miR423-3p, miR489, miR1971 🡱 | Not studied | Not adjusted |
| Gentamicin[25] | Rat  (Sprague–Dawley) | Urine  N=7:3 | Day 7 | Histopathology/KIM-1/ Beta-2-Microglobulin | | 375 | TaqMan® Low Density Array Cards/ next-generation sequencing | miR-378a-3p, miR-30e-3p, miR-125b-3p,miR-320-5p, miR-320-3p, miR-455-5p miR-100-5p, miR-21-3p, miR-21-5p 🡱  miR-203a-3p, miR-7641, miR-let-7d-5p, miR-3473, miR-5100🡳 | Not studied | Not adjusted |
| Gentamicin[32] | Rat  (Sprague–Dawley) | Blood  N=6:6 | Day 1 | SCr/  Histopathology | | 3 | TaqMan miRNA assays | miR-21 🡱 | Not studied | converting △Ct to linear 2^(△Ct)^ and multiplied by the fixed value (1000) and urine volume (ml) |
| Gentamicin[32] | Rat  (Sprague–Dawley) | Urine  N=6:6 | Day 1 | SCr/  Histopathology | | 3 | TaqMan miRNA assays | miR-21 and miR-155 🡳 | Not studied | converting △Ct to linear 2^(△Ct)^ and multiplied by the fixed value (1000) and urine volume (ml) |
| Melamine[22] | Rat  (F344 rats) | Serum  N=12:12 | Day 28 | SCr/  Histopathology | | 752 | Exiqon qPCR array/ TaqMan miRNA assays | miR-128-3p, miR-22-3p, miR-191a-3p, miR-210-3p 🡳 | Not studied | Not adjusted |
| N-phenylanthranylic acid[15] | Rat  (Wistar) | Urine  N=6:6 | 0, Day 1, 3, 6, 13, 20 and 27 | Histopathology | | 68 | FirePlex miRNA assay | miR-210-3p and 127-3p 🡱 | miR-210-3p and 127-3p correlated with OPN | Not adjusted |
| Paracetamol[23] | Human | Urine  N=43:61 | N/A | Histopathology | | 3 | SYBR Green- RT-qPCR | miR-21, miR-200c, miR-423 🡱 | Not studied | Not adjusted |
| Paracetamol[24] | Human | Plasma  N=38:30 | After NAC treatment | SCr | | 4 | SYBR Green PCR Kit/ miScript miRNA QC PCR Array | miR-122-5p, miR-885-5p, miR-151a-3p or miR-382-5p no change  miR-19a-3p, miR-19b-3p, miR-192-5p, miR34a- 5p and miR-3178 🡳 | Not studied | Not applicable |
| Puromycin[13] | Rat  (Sprague–Dawley) | Plasma  N=5:5 | Day 2, 3, 4, 5 and 7 | Histopathology | | 372 | Illumina MiSeq platform/TaqMan microRNA assays | miR-3473 🡱  miR-143-3p miR-122 🡳 | Not studied | Not applicable |
| Puromycin [25] | Rat  (Sprague–Dawley) | Urine  N=10:10 | Day 3, 9, and 16 | Histopathology | | 376 | TaqMan® Low Density Array Cards | miR-106a-5p, miR-223-3p, mi-17-5p, miR-218a-5p 🡱  miR-574, miR125a-3p, miR-196c-5p, miR-27b-3p and miR-30c-5p 🡳 | Not studied | Not adjusted |
| Puromycin[15] | Rat  (Wistar) | Urine  N=6:6 | 0, Day 1, 3, 6, 13, 20 and 27 | Histopathology | | 68 | FirePlex miRNA assay | miR-155-🡱 | miR-155-5p correlated with SCysC | Not adjusted |

, 🡱 and 🡳 represent up-regulated and down regulated miRNAs respectively. SCr-serum creatinine, KIM-1 - Kidney Injury Molecule-1, SCysC- serum cystatin C, OPN – Osteopontin, BNU - blood urea nitrogen, UCr – urinary creatinine,

uNAG - N-acetyl-β-glucosaminidase
